# Supplementary material for: Chromatin-prebound Crm1 recruits Nup98-HoxA9 fusion to induce aberrant expression of Hox cluster genes
Source: eLife. 2016 Jan 7;5:e09540. doi: 10.7554/eLife.09540 (PMC4718815; doi:10.7554/eLife.09540)
Supplement: Supplementary file 1. — DOI: http://dx.doi.org/10.7554/eLife.09540.024 [file elife-09540-supp1.docx]

| Gene/primer name | Forward (5' to 3') | Reverse (5' to 3') |  |
| --- | --- | --- | --- |
| *Oct4* | TAGGTGAGCCGTCTTTCCAC | GCTTAGCCAGGTTCGAGGAT | RT-qPCR |
| *Nanog* | TTGCTTACAAGGGTCTGCTACT | ACTGGTAGAAGAATCAGGGCT | RT-qPCR |
| *Gapdh* | AGGTCGGTGTGAACGGATTTG | TGTAGACCATGTAGTTGAGGTCA | RT-qPCR |
| *HoxA1* | GAGCTGCTTGGTGGTGAAAT | GCAGACCTTTGACTGGATGA | RT-qPCR |
| *HoxA3* | TCTTAACATGGAGGGAGCCA | TCTGAAGGCTACGTGTGCTG | RT-PCR |
| *HoxA4* | ggtggtgtacccctggatga | gacttgctgccgggtatagg | RT-PCR, RT-qPCR |
| *HoxA5* | CAGGGTCTGGTAGCGAGTGT | CTCAGCCCCAGATCTACCC | RT-PCR |
| *HoxA6* | GTCTGGTAGCGCGTGTAGGT | CCCTGTTTACCCCTGGATG | RT-PCR |
| *HoxA7* | CTTCTCCAGTTCCAGCGTCT | AAGCCAGTTTCCGCATCTAC | RT-PCR, RT-qPCR |
| *HoxA9* | ccacgcttgacactcacact | gctctcattctcggcattgt | RT-PCR, RT-qPCR |
| *HoxA10* | TCTTTGCTGTGAGCCAGTTG | CTCCAGCCCCTTCAGAAAAC | RT-PCR |
| *HoxA11* | cggccacactgaggacaag | aactctcgctccagctctcg | RT-PCR, RT-qPCR |
| *HoxB2* | cgaggtcggatcaccatcag | agctccagcagttgcgtgtt | RT-PCR |
| *HoxB7* | CTTTCTCCAGCTCCAGGGTC | AACTTCCGGATCTACCCCTG | RT-PCR |
| *HoxC6* | CAGGGTCTGGTACCGAGAGTA | TCCAGATTTACCCCTGGATG | RT-PCR |
| *β-Actin* | TTCCTTCTTGGGTATGGAAT | GAGCAATGATCTTGATCTTC | RT-PCR |
| *ChIP-HoxA1* | TCACTGAGTGATTGGATCCTGC | GGAGGAAGTGAGAAAGTTGGCAC | ChIP-qPCR |
| *ChIP-*  *HoxA2-HoxA3* | GTGGGGAGAGCAAATCCAAC | CGTCTACCCTCCTGCTCTTG | ChIP-qPCR |
| *ChIP-HoxA4* | CTCTGGAATAAAACGAAGGAGGC | GGACAAAGAATCAAAGGGCGAG | ChIP-qPCR |
| *ChIP-HoxA9* | ATCTGTATGCCTAGTCCCGCTCC | TTGATGTTGACTGGCGATTTTC | ChIP-qPCR |
| *ChIP-HoxA13* | CCTGTTGGTTCCAGGAGAAGTC | CCAAACTTCCCAGAGAAATGTCC | ChIP-qPCR |

Supplementary file 1. List of primers used in this study
